# Adiponectin Reverses the Hypothalamic Microglial Inflammation during Short-Term Exposure to Fat-Rich Diet

**DOI:** 10.3390/ijms20225738

**Published:** 2019-11-15

**Authors:** Hannah Lee, Thai Hien Tu, Byong Seo Park, Sunggu Yang, Jae Geun Kim

**Affiliations:** 1 Division of Life Sciences, College of Life Sciences and Bioengineering, Incheon National University, Incheon 406-772, Korea; 8973215@hanmail.net (H.L.); thaihientu@gmail.com (T.H.T.); bbs0808@naver.com (B.S.P.); 2 Department of Nano-Bioengineering, Incheon National University, Incheon 406-772, Korea; abiyang9@gmail.com; 3 Institute for New Drug Development, Division of Life Sciences, Incheon National University, Incheon 406-772, Korea

**Keywords:** energy metabolism, inflammation, hypothalamus, microglia, adiponectin

## Abstract

Adiponectin, an adipokine derived from the adipose tissue, manifests anti-inflammatory effects in the metabolically active organs and is, therefore, beneficial in various metabolic diseases associated with inflammation. However, the role of adiponectin in alleviating the hypothalamic inflammation connected to the pathogenesis of obesity has not yet been clearly interrogated. Here, we identified that the systemic administration of adiponectin suppresses the activation of microglia and thereby reverses the hypothalamic inflammation during short-term exposure to a high-fat diet. Additionally, we show that adiponectin induces anti-inflammatory effects in the microglial cell line subjected to an exogenous treatment with a saturated free fatty acid. In conclusion, the current study suggests that adiponectin suppresses the saturated free fatty acid-triggered the hypothalamic inflammation by modulating the microglial activation and thus maintains energy homeostasis.

## 1. Introduction

Inflammation is one of the major pathological causes implicated in the development of obesity and its related metabolic disorders [1,2,3]. Chronic inflammation triggered by over-nutrition results in perturbation of the activity of hypothalamic neurons, which govern the whole-body energy balance [4,5,6]. Recent studies report that microglia, the resident macrophages of the central nervous system (CNS), respond to the elevation of saturated free fatty acid (FFA) following consumption of the fat-rich diet and thereby trigger the hypothalamic inflammation [6]. This results in abnormal functioning of the neuronal circuit that controls the energy homeostasis.

Adiponectin, an adipokine derived from adipose tissue, has been reported as beneficial in alleviating multiple physiological disturbances [7,8,9]. It helps in alleviating a variety of metabolic diseases by ameliorating the cellular stresses including inflammation, oxidative stress, and endoplasmic reticulum stress [7,8,10,11,12]. Although, a recent study identified an anti-inflammatory effect of adiponectin in the CNS [11], the role of adiponectin in rectifying the hypothalamic inflammation during early over-nutrition period remains elusive. Therefore, this study was aimed at identification of the contribution of adiponectin in regulating the hypothalamic inflammation and the microglial function. We evaluated the inflammatory responses and microglial activation utilizing mice model treated for a short-term with fat-rich diet and a microglial cell line treated with a saturated FFA. In crux, the current study unravels the potential contribution of adiponectin in preventing the initiation of inflammatory response in the hypothalamic microglial cells in an early stage of obesity development.

## 2. Results

### 2.1. Adiponectin Reverses the Hypothalamic Inflammation Induced by Short-Term Exposure to High Fat Diet

**Figure 1:**
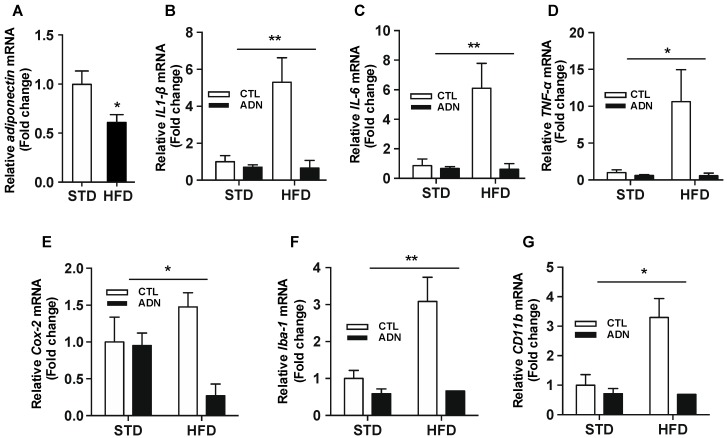
Systemic administration of adiponectin reverses the hypothalamic inflammation in response to short-term HFD treatment. C57BL/6 mice were fed either a Standard diet (STD) or a HFD for 4 weeks followed by i.p. injection of adiponectin or saline (as a control group) for 5 days. (**A**) The level of *adiponectin* mRNA in eWAT was significantly elevated 4 weeks after HFD treatment. The elevated mRNA levels of hypothalamic genes involved in inflammatory processes such as (**B**) *IL-1β*, (**C**) *IL-6*, and (**D**) *TNF-α* and (**E**) *Cox-2* observed in HFD-treated group were significantly reversed by i.p administration of adiponectin. Adiponectin treatment dampened the increase in the mRNA levels of hypothalamic (**F**) *Iba-1* and (**G**) *CD11b*, markers for microglia activation, in response to HFD treatment. Results are presented as mean ± SEM. *n* = at least 5 mice per group. * *p* < 0.05, ** *p* < 0.01 for effects of adiponectin on HFD-treated group versus effects of adiponectin on STD-treated group. CTL, control group; ADN, adiponectin-treated group.

In order to verify if adiponectin exerts anti-inflammatory effect in the hypothalamus during the early over-nutrition period, we evaluated the pattern of the hypothalamic inflammation after treatment with high fat diet (HFD) for 4 weeks combined with the systemic administration of globular adiponectin, which has a more potent effect on their identified functions [13]. In line with the previous finding that identified a reduction in circulating adiponectin level during short-term exposure to a fat-rich diet [13], we also confirmed a reduction in adiponectin mRNA level in epididymal white adipose tissue (eWAT) 4 weeks after high-fat diet treatment (Figure 1A). In accordance with the previous reports, we observed that HFD treatment resulted in a drastic increase in the expression levels of mRNA encoding inflammatory cytokines such as IL-1β (Figure 1B), IL-6 (Figure 1C), TNF-α (Figure 1D) as well as *Cox-2* gene (Figure 1E), the rate-limiting enzyme involved in the synthesis of the prostaglandin E2, which controls the cellular inflammatory process. This elevation of mRNA levels was effectively reversed by a systemic treatment with adiponectin for five days. Furthermore, we observed that an elevation in the levels of hypothalamic *Iba-1* and *CD11b mRNA*, the molecular markers of microglia, in HFD-treated group was almost completely rescued by the systemic treatment with adiponectin (Figure 1F,G). These findings suggest that adiponectin treatment exerts anti-inflammatory effects on the hypothalamus following short-term HFD exposure, at least in part, via targeting the microglial cells.

### 2.2. Adiponectin Suppresses the Microglial Activation Induced by Short-Term Exposure to HFD

**Figure 2:**
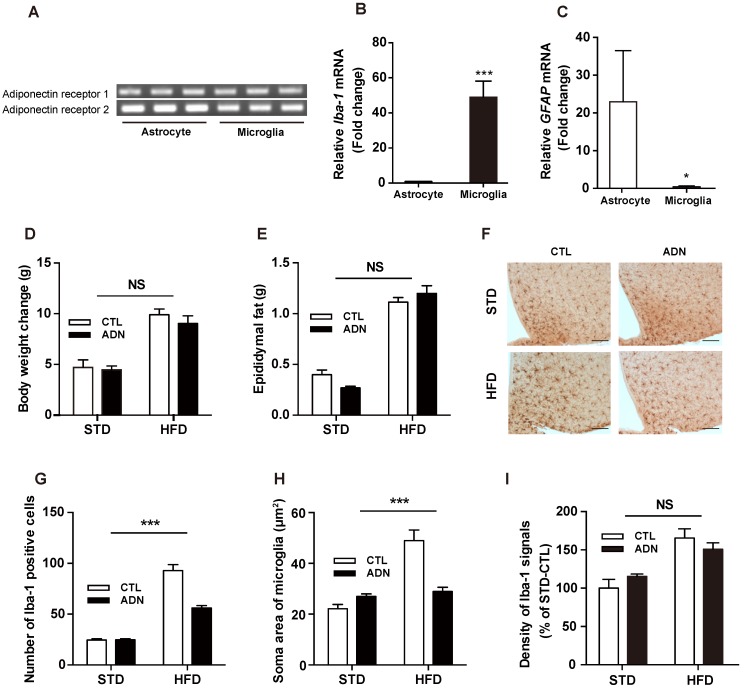
Adiponectin rescues the microglial activation caused by short-term exposure to high fat diet. (**A**) RT-PCR bands indicate the presence of AdipoR1 and AdipoR2 in both primary microglia and astrocytes. Confirmation of purification of microglia and astrocytes, as determined by levels of (**B**) *Iba-1* and (**C**) *GFAP* mRNAs. (**D**) Changes of body weight and (**E**) white adipose tissue weight seen in mice fed with either a STD or a HFD for 4 weeks followed by i.p. injection of adiponectin or saline (as a control group) for 5 days. (**F**) Representative images showing immunohistochemistry staining of Iba-1 in the sections of the hypothalamus from each group. Scale bar = 100 μm. The increased (**G**) number of microglial cells, and (**H**) soma area of microglia, observed in the hypothalamus of mice exposed to HFD were reversed by systemic adiponectin administration. (**I**) Adiponectin treatment did not alter the increased intensity of Iba-1 signals seen in the hypothalamus of mice fed a HFD. Results are presented as mean ± SEM. *n* = 5 mice per group. *** *p* < 0.001 for effects of adiponectin on HFD-treated group versus effects of adiponectin on STD-treated group or *Iba-1* mRNA level in astrocyte versus *Iba-1* mRNA level in microglia; * *p* < 0.05 for *GFAP* mRNA level in astrocyte versus *GFAP* mRNA level in microglia. NS, not significant; CTL, control group; ADN, adiponectin-treated group.

To determine whether adiponectin relieves the development of HFD-triggered hypothalamic inflammation by targeting the hypothalamic microglial cells, we identified the presence of adiponectin receptor 1 (*AdipoR1*) and adiponectin receptor 2 (*AdipoR2*) in the hypothalamic glial cells utilizing cultured primary microglia and astrocytes (Figure 2A). We also validated the purification of a single cell type determined by a strong expression of Iba-1, a molecular marker for microglia participating in membrane ruffling and phagocytosis in activated microglia (Figure 2B) [14], or GFAP, a molecular marker for astrocytes (Figure 2C). Based on the anti-inflammatory effects of adiponectin on hypothalamic inflammation triggered by short-term HFD treatment, we next performed immunohistochemistry (IHC) using an antibody against Iba-1 on brain slices from mice fed either a STD or a HFD combined with adiponectin to evaluate the impact of adiponectin treatment on microglia activation in the hypothalamus. We observed that the HFD treatment resulted in an increase in body weight (Figure 2D) and WAT weight (Figure 2E) as compared to the STD-treated group. On the contrary, the systemic administration of adiponectin for 7 days did not alter the increase in the body weight and WAT weight seen in the HFD-treated group (Figure 2D,E). Consistent with our cellular data, we observed that adiponectin treatment effectively reverses the microglial activation in the hypothalamus characterized by increased in a number of microglia (Figure 2F,G). In addition, we found that systemic treatment of adiponectin blocked the increase in microglia soma area in the HFD-treated hypothalamus (Figure 2H). However, adiponectin did not rescue the elevated of Iba-1 intensity seen in the HFD-treated hypothalamus (Figure 2I). These findings indicate that adiponectin reverses the effects of HFD on the hypothalamic inflammation by ameliorating the microglial activation during the early over-nutrition period.

### 2.3. Adiponectin Improves Palmitic Acid-Induced Inflammatory Responses in the Microglial Cells

**Figure 3:**
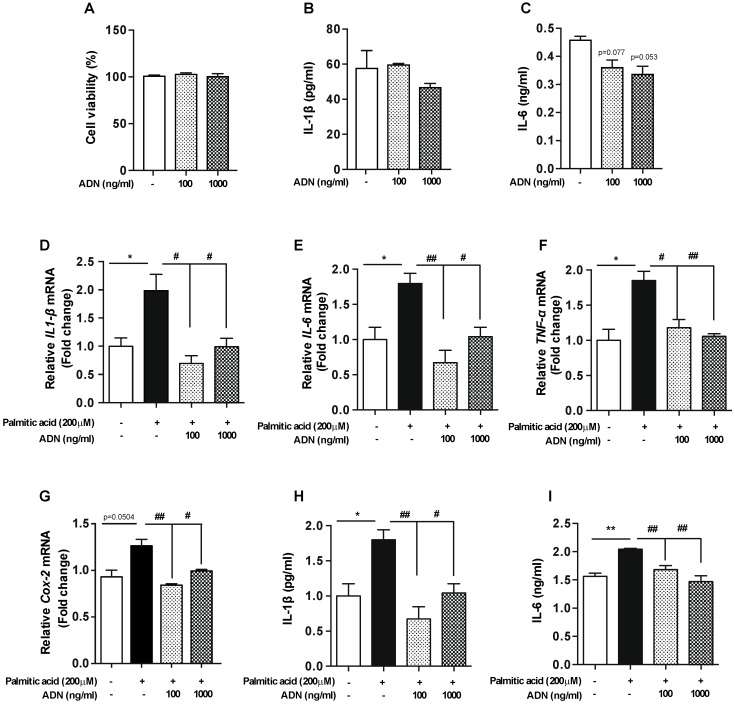
Adiponectin rescues the palmitic acid-induced inflammation in the microglial cell line. The BV-2 cells were seeded at a density of 5 × 10^5^ cells/well and pretreated with adiponectin at indicated concentration for 1 h followed by palmitic acid (200 µM) treatment for 4 h. Exogenous treatment of adiponectin did not change cell viability (**A**) or IL-1β release (**B**), but reduced IL-6 secretion (**C**). The palmitic acid-induced elevations of *IL-1β* (**D**), *IL-6* (**E**) *TNF- α* (**F**) and *Cox-2* (**G**) mRNA levels were reversed by adiponectin treatment in the BV-2 cells as determined by qPCR analysis. The palmitic acid-induced increase of IL-1β (**H**) and IL-6 (**I**) cytokine release was reversed by adiponectin treatment in the BV-2 cells as determined by ELISA. Results are presented as mean ± SEM. * *p* < 0.05 and ** *p* < 0.01 for palmitic acid-treated group versus vehicle-treated group; # *p* < 0.05 and ## *p* < 0.01 for palmitic acid + adiponectin (ADN)-treated group versus palmitic acid-treated group.

The consumption of fat-rich diet results in an elevation in the levels of circulating free fatty acids which cause the obesity pathogenesis linked to the hypothalamic inflammation [15,16]. Our previous study identified that the levels of saturated free fatty acid were elevated in both the hypothalamus and sera of mice fed a HFD for 4 weeks [17]. Therefore, to verify the anti-inflammatory role of adiponectin in alleviating the microglial inflammation induced by over-nutrition, we further evaluated the anti-inflammatory effect of adiponectin in the BV-2 microglial cell line by an administration of palmitic acid, a saturated free fatty acid followed by adiponectin treatment. We confirmed that single treatment of adiponectin did not alter cell viability (Figure 3A) or IL-1β release (Figure 3B) in cultured BV-2 microglial cells. However, adiponectin treatment led to a reduction in IL-6 secretion (Figure 3C). These findings indicated that adiponectin itself did not affect cellular inflammation and degeneration. In accordance with previous reports, palmitic acid treatment induced increase in the mRNA levels of inflammatory cytokines such as IL-1β (Figure 3D), IL-6 (Figure 3E) and TNF- α (Figure 3F), and *Cox-2* gene (Figure 3G) in cultured BV-2 microglial cells. This elevation of inflammatory responses triggered by palmitic acid was completely rescued by an exogenous adiponectin treatment. Moreover, the administration of adiponectin reversed the palmitic acid-induced release of IL-1β (Figure 3H) and IL-6 (Figure 3I) in the BV-2 microglial cells. These observations confirmed that adiponectin treatment reverses the development of hypothalamic inflammation and the associated microglial activation.

### 2.4. Adiponectin Reverses the Palmitic Acid-Induced Alterations in the Intracellular Signaling Molecules Involved in Inflammation

**Figure 4:**
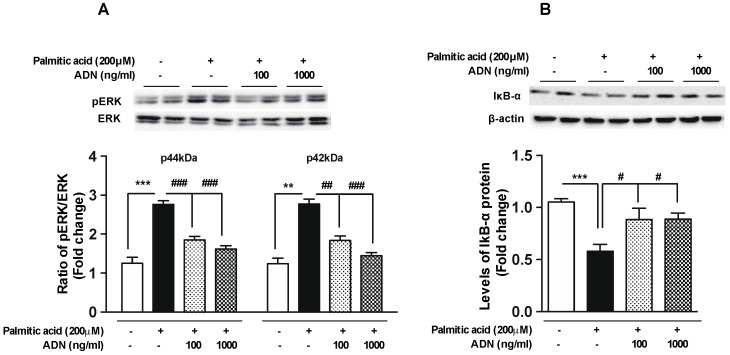
Adiponectin reverses palmitic acid-induced changes of the inflammatory mediators in the microglial cells. The BV-2 cells were seeded at a density of 1 × 10^6^ cells/well and pretreated with adiponectin for 1 h followed by a palmitic acid (200 µM) treatment for 30 min or 4 h. Palmitic acid-induced phosphorylation of ERK (**A**) and reduction of IkB-α synthesis (**B**) were reversed by adiponectin treatment at indicated concentration. Results are presented as mean ± SEM. ** *p* < 0.01 and *** *p* < 0.005 for palmitic acid-treated group versus vehicle-treated group; # *p* < 0.05, ## *p* < 0.01 and ### *p* < 0.005 for palmitic acid + adiponectin (AND)-treated group versus palmitic acid-treated group.

In order to further verify anti-inflammatory functions of adiponectin, we evaluated the phosphorylation of ERK, a molecular component of intracellular signal transduction in the inflammatory signaling pathway [18,19], and the level of IkB-α protein, which inhibits the activity of NF-κB transcription factor regulating the expression of multiple inflammatory cytokines [20]. As shown in the Figure 4, adiponectin reversed the palmitic acid-induced phosphorylation of ERK (Figure 4A) and degradation of IkB-α (Figure 4B). These observations suggest that adiponectin treatment suppresses the microglial inflammation in response to saturated FFA, at least in part, via modulating the general signaling molecules linked to the development of cellular inflammation.

## 3. Discussion

The present study highlights the beneficial effects of adiponectin in the alleviating the hypothalamic inflammation triggered by the over-nutrition that causes the disturbance in the functioning of hypothalamic neurons that regulate the whole-body energy metabolism.

Multiple lines of evidence have suggested that chronic inflammation in the hypothalamic neuronal circuit, which controls the appetite and energy expenditure, causes a variety of cellular stresses and thereby results in the energy imbalance [4,5,6]. The adipose tissue is a specialized connective tissue that functions as a major storage site for fats in the body [21,22,23]. However, it has been well established that the adipose tissue also acts as an endocrine organ besides regulating the energy balance [22,24,25]. The adipose tissue communicated with the hypothalamus by releasing multiple chemical messengers that systemically propagate the signals reflecting nutrient availability for the homeostatic control of energy homeostasis [21,22,26]. In addition, the adipose tissue also regulates the obesity pathogenesis by secreting a variety of inflammatory cytokines and adipokines that deteriorate the cellular stresses such as inflammation, endoplasmic reticulum stress, and oxidative stresses [21,22]. Although majority of the adipokines act as proinflammatory factors, adiponectin displays anti-inflammatory properties that potentially improve the dysfunction of metabolic controls in the body [8,27,28]. In the CNS, adiponectin inhibits neuronal degeneration by ameliorating the cellular stresses including inflammation [10,11,29].

A growing body of evidence suggested that the microglial cells essentially participates in the development of the neuronal inflammation [16,30,31] and the reactive gliosis is an important cellular event for both acute and chronic inflammation triggered by the over-nutrition [32,33]. Therefore, it is not surprising that the anti-inflammatory role of adiponectin in the microglial cells is coupled to the improvement of metabolic abnormalities. Since adiponectin binds via adiponectin receptors, AdipoR1 and AdipoR2 in the microglial cells, we confirmed the presence of these receptors in the microglial cells.

It has been quite well established that the short-term exposure of HFD results in an increased hypothalamic inflammation accompanied by the reactive gliosis of both microglia and astrocytes [5,6,34]. Intriguingly, mice having HFD for a couple of days displayed a significant increase in the inflammatory cytokines expression in the hypothalamus [5,6]. In line with this notion, we verified the role of adiponectin in ameliorating the hypothalamic inflammation during the consumption of the short-term fat-rich diet. Furthermore, we verified that adiponectin successfully reverses the inflammatory responses in the hypothalamic microglia during early over-nutrition period by observing the reduced microgliosis as determined by the accumulation of Iba-1 protein and the altered number and morphology of microglial cells.

A variety of substances are involved in the obesity-related pathogenesis. Among them, saturated FFAs are critical substances that trigger the inflammation in brain during early obesity period [16]. We observed that adiponectin effectively rescues the palmitic acid-induced microglial inflammation via regulation of the intracellular signaling molecules that mediate the inflammatory responses. Although there are substantial evidences highlighting the pathogenic factors associated with obesity and their impact on the development of metabolic dysfunction [35,36], the pathogenic substances involved in the initiation of hypothalamic inflammation during early over-nutrition period remain unexplored. Thus, it is also valuable to investigate the cellular and molecular responses following induction or prevention of the hypothalamic inflammation during early over-nutrition period. Indeed, previous studies indicated that adiponectin levels are slightly elevated during short-term exposure to HFD [37]. Therefore, we suggest that the anti-inflammatory effects of adiponectin might be due to the homeostatic response to suppress the hypothalamic inflammation induced by the multiple humoral factors such saturated FFAs and adipokines. However, the beneficial effects of adiponectin treatment on the severe obesity associated with the chronic inflammation need to be determined. Therefore, further studies are required to identify the beneficial effects of adiponectin in alleviating the cellular stresses following long-term treatment of adiponectin to the obesity model accompanied by chronic hypothalamic inflammation and the cellular lipotoxicity. Collectively, the current study identifies a reversible effect exerted by adiponectin treatment on the initiation of the hypothalamic inflammation during early over-nutrition period and provides novel insight into the strategies to prevent early disruption of the energy homeostasis.

## 4. Materials and Methods

### 4.1. Animals

**Figure 5:**
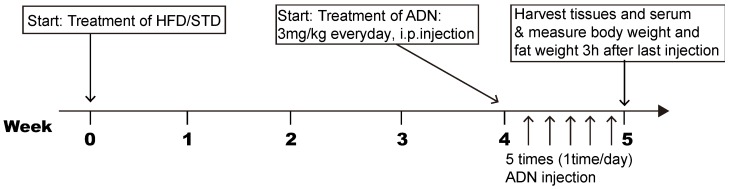
Flowchart showing the experimental procedure. Arrows indicate the start of treatment, sample preparation and analysis.

Seven-week-old C57B/L6 mice (Dae Han Bio Link, Eumseong, Korea) were maintained under specific pathogen-free conditions at 22 °C and given access to food and water ad libitum. To examine the effects of adiponectin on early obesity stage-induced hypothalamic inflammation, mice were adapted for a week, randomly divided into two groups and fed either a standard diet (STD, 10% calories from fat, Research Diet Inc., New Brunswick, NJ, USA) or a high-fat diet (HFD, 60% of calories from fat, Research Diets Inc.) for four weeks. The components of the HFD and STD are indicated in Table 1. For adiponectin treatment, mice were given intraperitoneal (i.p) injections of globular adiponectin (3 mg/kg, Lugen Sci, Bucheon, Korea) for five days. Brains and serum were collected 3 h after last injection. In addition, body weight and epididymal fat weight were measured when tissue was harvested. The experimental procedure is described in a flowchart (Figure 5). All the animal care and experimental procedures were performed in accordance with the protocols approved by the Institutional Animal Care and Use Committee (IACUC) at the Incheon National University (permission number: INU-2016-001).

### 4.2. Immunohistochemistry

Mice were anesthetized and perfused transcardially with 0.9% saline (*w*/*v*), followed by fixation with 4% paraformaldehyde in phosphate buffer (PB, 0.1 M, pH 7.4). Brains were isolated and post-fixed overnight with 4% paraformaldehyde PB buffer before the coronal sections (50 μm thickness) were prepared using a vibratome (5100 mz Campden Instruments; Leicestershire, UK). After washing in PB several times, the sections were preincubated with 0.3% Triton X-100 (Sigma-Aldrich, St. Louis, MO, USA) for 30 min at room temperature (RT) and then incubated with Iba-1 antibody (1:1000 dilution, Wako, Osaka, Japan) for overnight at RT. For diaminobenzidine- (DAB-) based Iba-1 IHC, sections were extensively washed and incubated with biotinylated anti-rabbit secondary antibody, ABC reagent (Vector Laboratories, Burlingame, CA, USA), and DAB substrate (Vector Laboratories).

### 4.3. Cell Culture and Treatments

The murine microglial BV-2 cells were maintained in Dulbecco′s modified Eagle medium (DMEM) with high glucose (Gibco BRL, Grand Island NY, USA), containing 5% (*v*/*v*) fetal bovine serum (Gibco BRL, Grand Island NY, USA) and incubated at 37 °C in humidified 5% CO_2_. For gene expression assay, cells were seeded at a density of 5 × 10^5^ cells/well in 12-well plate. After 24 h, the attached cells were pre-treated with adiponectin (100 ng/mL or 1000 ng/mL) for 1 h followed by the treatment with 200 µM palmitic acid (Sigma-Aldrich) for 4 h. The palmitic acid was dissolved in ethanol and conjugated into 10% bovine serum albumin (BSA).

### 4.4. Primary Astrocyte Culture

Following decapitation of five-day-old C57BL/6 mice, the diencephalon was removed under sterile conditions and triturated in Dulbecco’s modified Eagle′s medium (DMEM) F-12 containing 1% penicillin-streptomycin. The cell suspension was filtered through a 100-μm sterile cell strainer to remove debris and fibrous layers. The suspension was centrifuged, and the pellet resuspended in DMEM F-12, containing 10% fetal bovine serum (FBS) and 1% penicillin-streptomycin. The cells were then grown in this culture medium in 75-cm^3^ culture flasks at 37 °C and 5% CO_2_. When the cells reached confluence (at approximately nine days), microglia were separated from adhered astrocytes by shaking the culture at approximately 250 rpm for 2 h. The cells were then seeded onto 12-well tissue culture plates, previously coated with poly-d-lysine hydrobromide (50 μg/mL), after which they were distributed at 7.5 × 10^4^ cells/well and incubated at 37 °C with 5% CO_2_. For the astrocyte primary culture, the adhered cells were harvested using 0.05% trypsin-ethylenediamine tetraacetic acid, resuspended in DMEM F-12 containing 10% FBS and 1% penicillin-streptomycin, and centrifuged for 5 min at 1000 rpm. The cells were then seeded at a concentration of 5 × 10^5^ cells/mL in culture plates previously treated with poly-l-lysine hydrobromide (50 μg/mL), and grown for 24 h.

### 4.5. Quantitative Real-Time PCR (qRT-PCR)

Total RNA was isolated from the hypothalamus, cultured BV-2 cells, primary microglia and primary astrocytes, and reverse-transcribed to make cDNA using maxime RT PreMix kit (Intron Biotechnology, Seoul, Korea). Real-time PCR amplification of the cDNA was analyzed with SYBR Green Real-time PCR Master Mix (Toyobo Co., Ltd., Osaka, Japan) in a Bio-Rad CFX 96 Real-Time Detection System (Bio-Rad Laboratories, Hercules, CA, USA). The results were analyzed by the CFX Manager software and normalized to the housekeeping gene β-actin. Primer sequences used are *IL-1β* F-AGGGCTGCTTCCAAACCTTTGAC, R-ATACTGCCTGCCTGAAGCTCTTGT; *IL-6* F-CCACTTCACAAGTCGGAGGCTTA, R-GCAAGTGCATCATCGTTGTTCATAC; *TNF-α* F-TGGGACAGTGACCTGGACTGT, R-TTCGGAAAGCCCATTTGAGT; *Cox-2*F-TGCTGTACAAGCAGTGGCAA, R-AGGGCTTTCAATTCTGCAGCC; *Iba-1* F-AGCTTTTGGACTGCTGAAGG, R-TTTGGACGGCAGATCCTCATC; *CD11b* F-CCACTCATTGTGGGCAGCTC, R-GGGCAGCTTCATTCATCATGTC; *Adiponectin* F-GTTGCAAGCTCTCCTGTTCC, R-TCTCCAGGAGTGCCATCTCT; *GFAP* F-TCAATGACCGCTTTG CTAGC, R-ACTCGTGCAGCCTTACACAG; *β-Actin* F-TGGAATCCTGTGGCATCCATGAAAC, R-TAAAACGCAGCTCAGTAACAGTAACAGTCCG.

### 4.6. Measurement of Cytokine Levels

Supernatant from cultured BV-2 cells were collected 1 h after adiponectin (100 ng/mL or 1000 ng/mL) treatment followed by administration of palmitic acid (200 μM) for 24 h. The concentration of released cytokines was measured using mouse IL-1β and IL-6 DuoSet (R&D Systems, Minneapolis, MN, USA) according to the manufacturer′s instructions.

### 4.7. Immunoblot Analysis

BV-2 cells were seeded at a density of 1 × 10^6^ cells/well in 6-well plate and pre-treated with adiponectin (100 ng/mL or 1000 ng/mL) for 1 h followed by palmitic acid treatment for 30 min or 4 h. The cells were rinsed with phosphate buffered saline (PBS), followed by scraping and resuspension of the cell pellet in RIPA lysis buffer containing protease inhibitors, and centrifuged to remove debris, unbroken cells and cellular nuclei. The samples containing 20 μg of total protein were subjected to immunoblot analysis using polyclonal antibodies including anti-phospho-ERK, anti-ERK (Cell Signaling, Danvers, MA, USA), anti-IκBα (Santa Cruz Biotechnology, Santa Cruz, CA, USA), and anti-β-actin (Sigma-Aldrich, St. Louis, MO, USA). Quantification of band intensity was conducted using Image J software. The band intensities were normalized by dividing their values by the value for the total ERK protein, or β-actin protein, for the same sample on the same blot.

### 4.8. Statistical Analysis

Statistical analyses were performed by Prism 6.0 software (GraphPad Software, San Diego, CA, USA). All the data are expressed as mean ± SEM. An unpaired t test was performed to analyze the significance between the two experimental groups. Two-way ANOVA analysis was performed to detect the interaction between two treatments. Significance was taken at *p* < 0.05.

## Figures and Tables

**Table 1:** Components of the HFD and STD.

	High-Fat Diet (5.24 kcal/g)		Standard Diet (3.85 kcal/g)	
	g%	kcal%	g%	kcal%
Protein	26.2	20	19.2	20
Carbohydrate	26.3	20	67.3	70
Fat	34.9	60	4.3	10
	g	kcal	g	kcal
Casein80-mesh	200	800	200	800
L-Cystine	3	12	3	12
Cornstarch	0	0	315	1260
Maltodextrin 10	125	500	35	140
Sucrose	68.8	275.2	350	1400
Cellulose BW200	50	0	50	0
Soybean oil	25	225	25	225
Lard	245	2205	20	180
Mineral mix S10026	10	0	10	0
Dicalcium phosphate	13	0	13	0
Calcium carbonate	5.5	0	5.5	0
Potassium citrate	16.5	0	16.5	0
Vitamin mix V10001	10	40	10	40
Choline bitartrate	2	0	2	0
FD and C dye	0.05	0	0.05	0
